# Different criteria for body mass index classification for excess weight screening in children aged six to ten years

**DOI:** 10.1590/1984-0462/2024/42/2022132

**Published:** 2023-07-10

**Authors:** Alex Sander Freitas, Marise Fagundes Silveira, Desirée Sant Ana Haikal, Antônio Prates Caldeira, Vinícius Dias Rodrigues, Renato Sobral Monteiro-Júnior

**Affiliations:** a Universidade Estadual de Montes Claros, Montes Claros, MG, Brazil.

**Keywords:** Obesity, Overweight, Pediatric obesity, Nutritional status, Obesidade, Sobrepeso, Obesidade pediátrica, Status nutricional

## Abstract

**Objective:**

To evaluate the agreement between body mass index (BMI) parameters applied to children aged six to ten years in the city of Montes Claros (MG), Brazil with national and international criteria, also calculating their sensitivity and specificity regarding excess weight screening.

**Methods:**

A sample comprising 4151 children aged six to ten years was assessed, with height and body mass determined for BMI calculation. The obtained values were classified according to cutoff points established by the World Health Organization (WHO), International Obesity Task Force (IOTF), Centers for Disease Control and Prevention (CDC), Conde & Monteiro, and a recent local proposal. The agreement index between the mentioned criteria was calculated and thereafter the sensitivity and specificity.

**Results:**

The local proposal was proven to be highly consistent in most combinations, especially concerning the excess weight criteria of the World Health Organization (WHO) (k=0.895). Regarding excess weight, the local proposal presented sensitivity and specificity values of 0.8680 and 0.9956, respectively, indicating high BMI discrimination power.

**Conclusions:**

The locally applied BMI parameters for children aged six to ten years represent a valid, highly viable and practical proposal for excess weight screening in this population group, improving professional decision-making in their follow-up.

## RESUMO

**Objetivo:**

Analisar a concordância dos parâmetros de índice de massa corporal (IMC) para crianças com idade de seis a dez anos da cidade de Montes Claros/MG com os demais critérios de abrangência nacional e internacional, bem como sua sensibilidade e especificidade no rastreio do excesso de peso.

**Métodos:**

Foi utilizada uma amostra de 4.151 crianças de seis a dez anos, sendo mensuradas estatura e massa corporal para a determinação do IMC. Os valores obtidos foram classificados de acordo com os pontos de corte da World Health Organization (WHO), International Obesity Task Force (IOTF), Centers for Disease Control and Prevention (CDC), Conde & Monteiro e uma recente proposta local. Calculou-se o índice de concordância entre os critérios mencionados e, em seguida, a sensibilidade e a especificidade.

**Resultados:**

A proposta local mostrou-se altamente concordante na maioria das combinações, principalmente para o excesso de peso com a WHO (k=0,895). Com relação ao excesso de peso, a proposta local apresentou valores de 0,8680 e 0,9956 para a sensibilidade e especificidade respectivamente, mostrando alto poder de discriminação do IMC.

**Conclusões:**

Conclui-se que os parâmetros locais de IMC para crianças de seis a dez anos representam uma proposta válida, altamente viável e utilizável para o rastreio do excesso de peso desse grupo populacional, melhorando a tomada de decisão profissional no acompanhamento de tais indivíduos.

## INTRODUCTION

Childhood obesity has increasingly become a critical public health problem, reaching epidemic levels in several developed and developing countries.^1^ Characterized by excessive accumulation of body fat, obesity is considered a nutritional disorder resulting in increased body mass.^2^ It is related to several factors that may explain its establishment, such as socioeconomic level, sex and age, among others.^3^ Additionally, this condition is associated with or comprises in itself a risk factor for the development of other comorbidities such as type 2 diabetes, arterial hypertension, cardiovascular diseases, and certain types of cancer,^4^ contributing to increased morbidity and mortality rates in the general population, as many children and adolescents remain obese when adults.^5^

In this regard, according to the Global Burden of Disease Study,^6^ obesity rates have doubled in more than 70 countries since 1980, totaling 107.7 million obese children. In Brazil, data from the *Pesquisa de Orçamentos Familiares* (POF), carried out by the *Instituto Brasileiro de Geografia e Estatística* (IBGE), revealed a 34.8% rate of children between five and nine years of age presenting excess weight.^7^ In addition, a recent systematic review concerning studies performed in Brazil indicated a 14.1% obesity prevalence among Brazilian children and adolescents.^8^ In the city of Montes Claros (MG), data showed that 23.6% of children aged six to ten years are overweight.^9^

These results were obtained from Body Mass Index (BMI; body mass [kg]/height [m]^2^) assessments, the most frequently employed tool for excess weight screening in children and adolescents, as it is a non-invasive, accessible and easy-to-use alternative, suitable in both clinical practice assessments and in epidemiological studies.^9–11^

No universally accepted BMI classification criteria for children and adolescents is available so far, as this population group presents constant height and body mass fluctuations, typical of the growing process.^1,11^ This leads to a wide variation of BMI cutoff points according to age and sex, resulting in increasing proposals for criteria and studies on the accuracy of these specific child and adolescent age and sex parameters.^11–13^

Some criteria based on sample populations from several countries are noteworthy, such as those established by the World Health Organization (WHO)^14^ and the International Obesity Task Force (IOTF).^15^ Besides, other criteria have been applied to samples from specific regions, such as those established by the Centers for Disease Control (CDC)^16^ for North America, and Conde & Monteiro^17^ for the Brazilian population. It is important to note that each country or geographic region presents particular environmental, socio-behavioral, cultural and/or economic characteristics, and therefore, may present variations.^18^

The application of international or national references, depending on the territorial extension of each country, can result in distortions in study conclusions if the BMI of a given population does not exhibit the same characteristics as the one used as reference.^19^ Consequently, it has been widely recognized that child growth is determined by genetic and environmental factors and that it is mandatory for each country to apply specific curves to a given population to assess growth patterns and nutritional status.^20^ This is particularly factual concerning overweight and obesity patterns intrinsically associated with socio-environmental contexts.

In this context, a specific proposal for BMI cutoff has been previously developed for children aged six to ten years in Montes Claros (MG), Brazil.^21^ Thus, the aim of the present study was to assess agreements between different BMI classification criteria employed worldwide and calculate their sensitivity and specificity regarding overweight and obesity screenings in children aged six to ten years.

## METHOD

This study comprises a cross-sectional assessment (one arm) performed according to the Strengthening the Reporting of Observational Studies in Epidemiology (STROBE) standards.^22^

The study population consisted of 30,625 schoolchildren aged 6.0 to 10 years enrolled in the first grades of elementary school in Montes Claros (MG), Brazil. A total of 248 elementary schools are located in the municipality. The sampling process occurred according to clusters, with 16 schools being drawn. Seventy students of each age group from 6.0 to 10 years were randomly selected through a simple draw among the participating schools, comprising 35 boys and 35 girls.

The sample size was established with an error of three percentage points and a confidence interval of 95% (95%CI), a design effect (Deff) of 1.5, plus 10% to account for possible losses and/or refusals. Thus, a total of 4480 children were selected, 329 of which were excluded for not delivering the Free and Informed Consent Term (FICT) signed by their parents/tutors or due to their absence at the time of data collection. The final sample consisted of 4,151 students, 2119 boys and 2032 girls.

The project was approved by the Ethics Committee in Research with Human Beings under protocol no. 798.138 of the State University of Montes Claros (Unimontes). Afterward, a letter of clarification alongside an authorization request was sent to the Montes Claros Municipal Department of Education. After obtaining all authorizations, a letter with the same content was delivered to the principal of each selected school before data collection began. In addition, parents/tutors were informed of the study procedures and objectives, according to the National Health Council resolution 466/12.

Parents/tutors signed the FICT authorizing their child’s participation. Subsequently, each school was visited, and data collection was performed during Physical Education classes.

Anthropometric variables were determined according to Lohman et al.^23^ The digital scale by Welmy^®^, Brazil (0.1 kg precision) and a coupled stadiometer (0.1 cm precision) were used for collecting body mass and height measures, and thereafter the BMI was calculated.

The data were entered and analyzed using the Statistical Package for Social Sciences (SPSS^®^) version 24.0, software for Windows by IBM, United States of America (USA). Descriptive statistical procedures were initially applied, i.e, minimum, maximum, mean and standard deviations of the variables for sample characterization. Overweight and obesity rates were determined along their respective 95%CI according to the BMI classification criteria of the WHO,^14^ IOTF,^15^ CDC,^16^ Conde and Monteiro^17^, and the local Brazilian proposal developed by Freitas et al.^21^

The *Kappa* agreement index (k) was applied to verify agreement between the assessed criteria, adopting a significance level of p≤0.05. Overweight and obesity sensitivity and specificity estimates for each BMI cutoff classification were calculated employing the WHO proposal^14^ as reference, compared to IOTF^15^, CDC^16^, Conde and Monteiro^17^ and Freitas et al.^21^ criteria.

The cutoff points used by the WHO^14^ were extracted from data of a study conducted in six countries: Brazil, Ghana, India, Norway, Oman, and the USA. The figures published by IOTF^15^ are derived from six significant studies conducted in Brazil, the United Kingdom, Hong Kong, the Netherlands, Singapore, and the USA. The CDC^16^ growth references values are based on data from five nationally representative surveys of young North Americans, while the Brazilian proposal by Conde & Monteiro^17^ was elaborated with data from the Brazilian population.

The local proposal developed by Freitas et al.^21^ used a sample of 3863 schoolchildren aged between six and ten years of both sexes, from Montes Claros (MG), using the Lambda Mu and Sigma (LMS) statistical method, which consists of the Box-Cox transformation to normalize data; new BMI cutoff values were established for that population.

For the calculation and analysis of sensitivity and specificity with a 95%CI, the participants were grouped as

1. Overweight: eutrophic and excess weight individuals;
2. Obesity: only obese individuals; and
3. Excess weight: overweight added to obesity cases.

## RESULTS

A total of 4151 children aged 6 to 10 years from Montes Claros (MG), Brazil were evaluated, comprising 2119 boys and 2032 girls. Table 1 displays the descriptive values of the sample characteristics, both by sex and grouped. Table 2 presents the determined overweight and obesity rates at a 95%CI according to WHO,^14^ IOTF,^15^ CDC,^16^ Conde and Monteiro,^17^ and the local Freitas et al.^21^ criteria.

**Table 1.** Sample characterization according to age, height, body mass and Body Mass Index.

	Minimum	Maximum	Mean	Standard deviation
Male (n=2119)				
Age (years)	6.0	10	7.96	1.13
Height (cm)	99.5	160.8	131.46	10.07
Body mass (kg)	15.3	64.5	29.06	6.92
BMI	10.61	31.60	16.65	2.51
Female (n=2032)				
Age (years)	6.0	10	7.96	1.13
Height (cm)	102.3	160.4	132.03	10.76
Body mass (kg)	15.4	62.4	29.28	7.36
BMI	10.76	33.63	16.60	2.61
Total (n=4151)				
Age (years)	6.0	10	7.96	1.13
Height (cm)	99.5	160.8	131.74	10.42
Body mass (kg)	15.3	64.5	29.17	7.14
BMI	10.61	33.63	16.63	2.56

BMI: Body Mass Index (body mass [kg]/height [m]^2)^.

**Table 2.** Overweight, obesity and excess weight rates according to sex and different Body Mass Index (BMI) classification criteria.

Criterion	Sex	Overweight		Obesity		Excess weight	
		(%)	95%CI	(%)	95%CI	(%)	95%CI
WHO^14^	Male	16.0	14.50–17.50	16.9	15.47–18.33	32.9	29.97–35.83
	Female	13.7	12.20–15.20	12.3	10.87–13.73	26.0	23.07–28.93
	Total	14.9	13.82–15.98	14.6	13.53–15.67	29.5	29.20–31.65
IOTF^15^	Male	16.7	15.09–18.31	4.0	3.12–4.88	20.7	18.21–23.19
	Female	16.5	14.89–18.11	4.3	3.42–5.18	20.8	18.31–23.29
	Total	16.6	15.47–17.73	4.2	3.59–4.81	20.8	19.06–22.54
CDC^16^	Male	17.9	16.28–19.52	8.1	7.05–9.15	26.0	23.33–28.67
	Female	16.6	14.98–18.22	6.2	5.15–7.25	22.8	20.13–25.47
	Total	17.2	16.05–18.35	7.2	6.41–7.99	24.4	22.45–26.34
Conde and Monteiro^17^	Male	19.5	17.67–21.33	3.0	1.91–4.09	22.5	19.58–25.42
	Female	23.0	21.17–24.83	6.7	5.61–7.79	29.7	26.78–32.62
	Total	21.2	19.96–22.44	4.8	4.15–5.45	26.0	24.11–27.89
Freitas et al.^21^	Male	13.1	11.69–14.51	14.3	12.86–15.74	27.4	24.55–30.25
	Female	12.0	10.59–13.41	12.5	11.06– 13.94	24.5	21.65–27.35
	Total	12.6	11.59–13.61	13.4	12.36–14.44	26.0	23.95–28.05

WHO: World Health Organization; IOTF: International Obesity Task Force; CDC: Centers for Disease Control and Prevention.

Table 3 presents the agreement values (*Kappa* index) between the applied overweight, obesity and excess weight criteria. All values were significant at p<0.01, and the highest overweight agreements were observed between the IOTF and CDC criteria at 0.815, and between the WHO and Freitas et al.,^21^ at 0.803. Concerning obesity and excess weight, the highest agreement values were observed between the WHO^14^ and Freitas et al.,^21^ criteria at 0.893 and 0.895 respectively, demonstrating a strong agreement between them.

**Table 3.** Agreement (*Kappa* and standard error) between the Body Mass Index classification criteria for overweight, obesity and excess weight.

	Overweight k (p)	Obesity k (p)	Excess weight k (p)
WHO^14^ x IOTF^15^	0.519 (0.021)	0.404 (0.022)	0.754 (0.012)
WHO^14^ x CDC^16^	0.715 (0.017)	0.619 (0.019)	0.847 (0.009)
WHO^14^ x Conde and Monteiro^17^	0.663 (0.018)	0.453 (0.021)	0.812 (0.010)
WHO^14^ x Freitas et al.^21^	0.803 (0.014)	0.893 (0.010)	0.895 (0.008)
IOTF^15^ x CDC^16^	0.815 (0.013)	0.715 (0.024)	0.864 (0.009)
IOTF^15^ x Conde and Monteiro^17^	0.769 (0.013)	0.795 (0.023)	0.811 (0.011)
IOTF^15^ x Freitas et al.^21^	0.684 (0.019)	0.619 (0.023)	0.838 (0.010)
CDC^16^ x Conde and Monteiro^17^	0.770 (0.013)	0.702 (0.024)	0.826 (0.010)
CDC^16^ x Freitas et al.^21^	0.779 (0.016)	0.663 (0.019)	0.880 (0.009)
Conde and Monteiro^17^ x Freitas et al.^21^	0.753 (0.016)	0.490 (0.022)	0.860 (0.009)

All *Kappa* coefficients were significant (p<0.01). WHO: World Health Organization; IOTF: International Obesity Task Force; CDC: Centers for Disease Control and Prevention.

Table 4 displays the calculated overweight, obesity and excess weight sensitivity and specificity values compared to the WHO criteria^14^. All criteria exhibited high specificity for the overweight diagnosis, with values ranging from 0.9713 to 0.9959. Strong results on obesity and excess weight were also observed, ranging from 0.9921 to 1.000 and 0.9646 to 0.9959, respectively. Regarding sensitivity, the values of overweight, obesity and excess weight ranged, respectively, from 0.4055 to 0.7264, 0.2845 to 0.8701 and 0.6927 to 0.8680. In this case, the local criterion proposed by Freitas et al.^21^ was more sensitive than the other investigated criteria.

**Table 4.** Sensitivity and specificity of different Body Mass Index classification criteria for determining overweight, obesity and excess weight conditions with the World Health Organization classification as reference.

Overweight – WHO^14^					
Criterion		Yes	No	Sensibility [95%CI]	Specificity [95%CI]
IOTF^15^	Yes	251	12	0.4055 [0.3675–0.4446]	0.9959 [0.9928–09977]
	No	368	2912		
CDC^16^	Yes	386	19	0.6246 [0.5858–0.6619]	0.9935 [0.9899–0.9958]
	No	232	2905		
Conde and Monteiro^17^	Yes	390	84	0.6311 [0.5923–0.6682]	0.9713 [0.9646–0.9757]
	No	228	2840		
Freitas et al.^21^	Yes	430	12	0.7264 [0.6891–0.7607]	0.9959 [0.9928–0.9976]
	No	162	2911		
**Obesity – WHO^14^**					
IOTF^15^	Yes	173	0	0.2845 [0.2501–0.3217]	1.0 [0.9989–1.0]
	No	435	3543		
CDC^16^	Yes	297	1	0.4885 [0.4490–0.5282]	0.9997 [0.9984–1.0]
	No	311	3542		
Conde and Monteiro^17^	Yes	199	1	0.3273 [0.2912–0.3656]	0.9997 [0.9984–1.0]
	No	409	3542		
Freitas et al.^21^	Yes	529	28	0.8701 [0.8410–0.8945]	0.9921 [0.9886–0.9945]
	No	79	3515		
**Excess weight – WHO^14^**					
IOTF^15^	Yes	850	12	0.6927 [0.6664–0.7179]	0.9959 [0.9928–0.9977]
	No	377	2912		
CDC^16^	Yes	995	19	0.8109 [0.7881–0.8318]	0.9935 [0.9899–0.9958]
	No	232	2905		
Conde and Monteiro^17^	Yes	997	84	0.8126 [0.7898–0.8334]	0.9646 [0.9589–0.9767]
	No	230	2840		
Freitas et al.^21^	Yes	1065	13	0.8680 [0.8479–0.8858]	0.9956 [0.9924–0.9974]
	No	162	2911		

WHO: World Health Organization); IOTF: International Obesity Task Force; CDC: Centers for Disease Control and Prevention.

## DISCUSSION

The present study found a high sensitivity and specificity of the values proposed by Freitas et al.^21^ for the classification of BMI with regard to overweight, obesity and excess weight for children aged six to ten years from Montes Claros (MG). In addition, significant values of agreement were also found with the cutoff points of the WHO,^14^ IOTF,^15^ CDC^16^ and Conde and Monteiro^17^ proposals. Such findings lead to the consideration of regional BMI cutoff for children and adolescents.

Monitoring childhood overweight and obesity rates is paramount for the disease control and the consequent development of action strategies by public health agencies.^4^ Several studies on overweight and obesity rates in children and adolescents have been published in the scientific literature in recent years, nevertheless, the variability of BMI classification criteria and the divergences regarding nutritional status diagnoses are still complicating factors.^12,13^

Confirming this premise, Dinsdale et al.^24^ highlighted that numerous pediatric obesity definitions have been established by various specialized organizations, such as the WHO,^14^ IOTF^15^ and CDC,^16^ making childhood obesity definitions even more challenging. In regards to Brazil, a proposal was also put forth by Conde and Monteiro^17^ employing specific BMI cutoff points for children and adolescents.

These notable criteria variabilities were assessed in a systematic review presented by Jansen et al.,^10^ who investigated different strategies for the diagnosis of childhood obesity. The results indicated that the most applied BMI reference system was based on IOTF^15^ criteria, observed in 37% of the selected review articles, followed by CDC^16^ and WHO^14^ curves (29.6% and 14.8%, respectively), and 18.5% considered specific reference curves for the countries in which they were carried out, such as the Conde & Monteiro criteria applied in Brazil. A more recent systematic review performed in Brazil^25^ assessed a total of 40 articles produced in 2018 and 2019 and pointed out that 32 studies applied WHO^14^ cutoff points, five used the IOTF^15^ criteria, two applied CDC^16^ values, and only one employed the Count and Monteiro^17^ criteria. These studies reflect a reality that cannot be ignored regarding the number of criteria employed to classify BMI in children and adolescents worldwide; and several studies have compared different cutoff points searching for the most suitable criteria.

Studies started to apply more than one reference criterion for child BMI calculation, also carrying out comparisons, agreement analyses and sensitivity and specificity calculations for each criterion. For example, Barbosa Filho et al.^13^ conducted a study in 2010 with the aim of verifying the agreement values between WHO^14^ criterion and the IOTF,^15^ CDC^16^ and Conde and Monteiro^17^ proposals for a sample of 619 children aged six and seven years in Fortaleza (CE), Brazil. The authors reported a very good agreement between the proposed WHO^14^ guidelines and IOTF^15^ and CDC^16^ values (k=0.82), as well as for the cutoff points proposed by Conde and Monteiro^17^ (k=0.68). Unlike the present study, those authors did not verify agreements between all criteria; they adopted the WHO criterion as a reference, and did not present specific nutritional status values. Considering the excess weight condition, the values reported herein were k=0.754, k=0.847, and k=0.812 for the IOTF,^15^ CDC^16^ and Conde and Monteiro^17^ criteria, respectively. These differences can be explained by the applied child age range and the different sample sizes between the studies.

Duarte et al.^26^ also reported agreement values between the same criteria of the previously mentioned study in the state of Amazonas, Brazil, although assessing only 1,387 children aged two to six years. In that case, a k value of 0.736 was calculated between the WHO^14^ and Conde and Monteiro^17^ criteria, k=0.610 between the IOTF and Conde and Monteiro, and k=0.492 for the WHO and IOTF criteria. In another Brazilian study performed in southern Brazil, 1,715 children and adolescents aged 10 to 17 years were investigated and agreement concerning excess weight was assessed between the WHO and CDC criteria, with an agreement of k=0.743,^27^ lower than that reported in the present study (k=0.847).

Still concerning Brazil, in the state of Santa Catarina, another study^28^ aimed to verify the sensitivity and specificity of the cutoff points proposed by the WHO and by Conde and Monteiro to assess excess weight in a sample comprising 2,795 children aged seven to ten years. In this case, sensitivity and specificity values of 0.925 and 0.759 were obtained, respectively, for the WHO^14^ criteria, and 0.986 and 0.850 for sensitivity and specificity, respectively, for the Conde and Monteiro^17^ cutoff points.

As for the present study, the sensitivity values of all analyzed criteria were considerably lower, except for the new BMI parameters proposed by Freitas et al.,^21^ which presented significantly high sensitivity (0.8680) and specificity (0.9956) values for the excess weight condition, as it constitutes a specific proposal for the northern Minas Gerais population, while the other cited studies applied both national and international criteria.

The applied logic of comparing different strategies for nutritional status diagnosis through the BMI is also noted internationally. For example, in Italy, Valerio et al.^29^ assessed the behavior of the curve proposed by the Italian Society of Pediatric Endocrinology and Diabetology (ISPED) in comparison to WHO^14^ and IOTF^15^ reference curves; they used a sample comprising 6,070 children and adolescents aged 5 to 17 years, that reported k=0.900 for the overweight condition according to the ISPED in relation to the other studied criteria. Concerning obesity, the calculated agreements in relation to the ISPED were k=0.664 for the WHO and k=0.875 for the IOTF.

In the present study, the agreements between the WHO^14^ and IOTF^15^ criteria compared to the local proposal established by Freitas et al.,^21^ presented quite different values. The local proposal, agreements with the WHO^14^ were very good for overweight and obesity (k=0.803 and k=0.893, respectively), but lower for the IOTF^15^ values (k=0.684 and k=0.619, respectively), demonstrating a high agreement with WHO^14^ criteria for the assessment of the nutritional status of the population studied in Montes Claros in northern Minas Gerais, Brazil.

Still in comparison with the Italian study,^29^ the authors also proposed to verify the sensitivity and specificity of the Italian criteria for the overweight and obesity diagnoses, obtaining sensitivity and specificity values of 0.9810 and 0.2200, respectively, for the overweight condition, and 0.8630 and 0.4130, respectively, for obesity. In relation to the proposal by Freitas et al.^21^ values were very different, calculated as 0.7264 for sensitivity and 0.9959 for specificity in the case of the overweight condition, and 0.8701 and 0.9921, respectively, for the obesity condition. In this sense, the criterion proposed by Freitas et al.^21^ presents both adequate sensitivity and specificity for the detection of overweight and obesity conditions for the proposed population. Furthermore, the Italian study^29^ is a national proposal, unlike Freitas et al.,^21^ which comprises a regional proposal.

In another study carried out in Iran,^30^ the national proposal for the classification of BMI in Iranian children and adolescents was compared to the WHO^14^ and CDC^16^ cutoff points. The authors used a sample comprising 22,718 children and adolescents aged 6 to 18 years and compared the percentiles for the low weight (5^th^), normal weight (50^th^), overweight (85^th^), and obesity (95^th^) classifications. They reported significant differences (p<0.05) in the low weight percentiles for both sexes, suggesting that Iranian children exhibit a lower BMI than the reference population used in other established cutoff points. In addition, the values referring to the 95^th^ percentile, which determines obesity in the Iranian proposal, were very close to the WHO^14^ values but significantly lower (p<0.05) than the 95^th^ percentile of the CDC^16^ values. Thus, epidemiological studies performed in Iran employing the CDC proposal may have underestimated the prevalence of obesity in the Iranian pediatric population.

Such findings reinforce the discussion on the use of international references in nutritional status diagnoses in children and adolescents worldwide. Genetic, ethnic, environmental, and sociodemographic variations must be considered when applying BMI cutoff points in different countries.^30^ The findings of the Iranian study are consistent with the present study, which also detected differences among BMI classifications according to the most employed references, including the national one.

Within this context, regional differences in countries as large as Brazil, displaying continental dimensions, cannot be neglected, as geographic, cultural, economic, and ethnic characteristics are striking. As a limitation of the present study, we highlight the restricted age range of the participants, from six to ten years old. On the other hand, this assessment presents a significant contribution in terms of using BMI cutoff points for specific children and adolescents per region, as national and international references can lead to results that do not represent the real situation of a given location.

In conclusion, the comparison carried out herein among the different BMI classification proposals for the nutritional status of children aged six to ten years in Montes Claros (MG), Brazil, demonstrates that the local proposal developed by Freitas et al.^21^ exhibits high agreement levels with both national and international guidelines. Furthermore, it is also proven as more sensitive and as specific or more for the detection of overweight, obesity and excess weight in relation to other references employing the WHO^14^ proposal as a standard.

Therefore, the use of the proposed BMI cutoff points from a regional perspective was proven valid and viable due to high calculated sensitivity and specificity values, in addition to the agreement with the other references; for this reason, we recommend the establishment of cutoff points covering the entire childhood and adolescence period for specific regions.

## Data Availability

The database that originated the article is available with the corresponding author.
